# Loss of Rictor with aging in osteoblasts promotes age-related bone loss

**DOI:** 10.1038/cddis.2016.249

**Published:** 2016-10-13

**Authors:** Pinling Lai, Qiancheng Song, Cheng Yang, Zhen Li, Sichi Liu, Bin Liu, Mangmang Li, Hongwen Deng, Daozhang Cai, Dadi Jin, Anling Liu, Xiaochun Bai

**Affiliations:** 1 Academy of Orthopedics in Guangdong Province, The Third Affiliated Hospital of Southern Medical University, Guangzhou 510630, China; 2 State Key Laboratory of Organ Failure Research, Department of Cell Biology, School of Basic Medical Sciences, Southern Medical University, Guangzhou 510515, China; 3 Department of Biochemistry, Institute of Genetic Engineering, Southern Medical University, Guangzhou 510515, China; 4 Department of Spine Surgery, The Third Affiliated Hospital, Sun Yat-Sen University, Guangzhou 510630, China

## Abstract

Osteoblast dysfunction is a major cause of age-related bone loss, but the mechanisms underlying changes in osteoblast function with aging are poorly understood. This study demonstrates that osteoblasts in aged mice exhibit markedly impaired adhesion to the bone formation surface and reduced mineralization *in vivo* and *in vitro*. Rictor, a specific component of the mechanistic target of rapamycin complex 2 (mTORC2) that controls cytoskeletal organization and cell survival, is downregulated with aging in osteoblasts. Mechanistically, we found that an increased level of reactive oxygen species with aging stimulates the expression of miR-218, which directly targets Rictor and reduces osteoblast bone surface adhesion and survival, resulting in a decreased number of functional osteoblasts and accelerated bone loss in aged mice. Our findings reveal a novel functional pathway important for age-related bone loss and support for miR-218 and Rictor as potential targets for therapeutic intervention for age-related osteoporosis treatment.

Age-related bone loss is a primary cause of osteoporotic fractures in the elderly population, characterized predominantly by reduced bone formation in the setting of persistent bone resorption.^1, 2, 3, 4^ Age-related osteoblast dysfunction is thought to be a major cause of age-related bone loss in both men and women beyond the fifth decade. However, the cellular and molecular mechanisms underlying changes in osteoblast function with aging are poorly understood.^5, 6, 7^

The osteoblast is a unique bone-forming cell derived from bone marrow mesenchymal stem cells (BMSCs), which are pluripotent cells that give rise to different tissue-specific cells including osteoblasts, chondrocytes and adipocytes.^8, 9^ Because age-related osteopenia is characterized by reduced bone formation and increased marrow fat accumulation, it has been suggested that the excessive accumulation of marrow adipocytes following bone loss is caused by unbalanced differentiation of BMSCs into marrow adipocytes in excess of osteoblasts.^4, 5, 6^ Most previous studies focused on the molecular mechanisms behind the switch from osteoblast to adipocyte differentiation in BMSCs during aging.^10, 11, 12, 13^ However, bone formation is dependent on the number and the activity of individual osteoblasts recruited at bone formation sites, and the functional activity of osteoblasts during *in vivo* bone formation depends not only on the number and generation rate of osteoblastic cells but also on their functional lifespan.^14, 15, 16^ The contribution to aged-related bone loss of age-related changes in the migration, adhesion, mineralization and apoptosis of osteoblasts are currently understudied.^7, 17^ In addition, the signaling pathways that control these processes remain largely unknown.

The evolutionarily conserved mechanistic target of rapamycin (mTOR) forms two functionally distinct complexes. The first, mTOR complex 1 (mTORC1), consisting of mTOR, Raptor and mLST8 (GβL), is sensitive to rapamycin and thought to control autonomous cell growth in response to nutrient availability and growth factors.^18, 19, 20^ The second complex, mTORC2, containing the core components mTOR, mSIN1, mLST8 and Rictor, is largely insensitive to rapamycin. mTORC2 was discovered more recently, and limited information is available on its regulation and function. Phosphorylation of Akt at the hydrophobic motif site (Ser473) is necessary for the activity towards some (but not all) substrates, and is the best-characterized readout of mTORC2 activity. Studies so far suggest that mTORC2 specifically senses growth factors to regulate cell proliferation, actin cytoskeleton organization and cell survival.^19, 21, 22, 23^

Several recent studies have demonstrated that deletion or downregulation of Rictor/mTORC2 in BMSCs resulted in reduced osteogenic differentiation capacity and enhanced adipogenic differentiation potential.^24, 25, 26^ mTORC2 signaling regulates skeletal growth and lineage selection in MSCs through unknown mechanisms.^24, 25, 26^ Its role in age-related bone loss and the underlying upstream and downstream regulatory mechanisms have not been reported to our knowledge. In this report, we demonstrate that mice with osteoblast-specific Rictor deletion exhibited accelerated age-related bone loss, and identified a novel mechanism by which the increased level of reactive oxygen species (ROS) with aging stimulates miR-218 expression, which directly targets Rictor and reduces osteoblast adhesion and survival, resulting in age-related bone loss.

## Results

### Osteoblast adhesion and survival are markedly reduced and Rictor expression is downregulated with aging *in vitro* and *in vivo*

**Figure 1:**
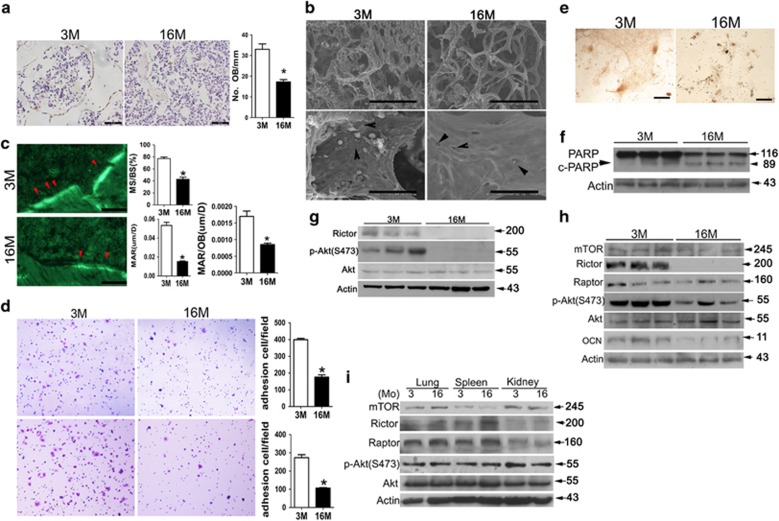
Osteoblast adhesion and survival are markedly reduced and Rictor expression is downregulated with aging *in vitro* and *in vivo*. (**a**) Representative immunohistochemical (IHC) staining for osteocalcin (OCN) from 3-month-old and 16-month-old mice (*n*=6). Arrow, osteoblast; BS, bone surface; scale bar, 50 *μ*m; *P<0.01 *versus* 3M. (**b**) SEM image of femora trabecular bones from 3- and 16-month-old mice. Upper panel showed less and thinner trabecula bone in 16-month-old mice. Black arrow, osteoblasts or osteocytes, up panel scale bar, 500 *μ*m; lower panel scale bar, 50 *μ*m. (**c**) Photomicrographs of fluorescent calcein staining of 3- and 16-month-old mice and quantification of mineralizing surface/bone surface (MS/BS), mineral apposition rate (MAR) and MAR/osteoblast. Arrow: bone formation surface; scale bar, 20 *μ*m (*n*=3 *P<0.01 *versus* 3M). (**d**) Cell adhesion analyses of calvarial osteoblast cultures from 3- and 16-month-old mice with (lower panel) or without (upper panel) matrigel in the plate (*n*=3 *P<0.01 *versus* 3M). (**e**) Representative photomicrographs of bone nodule formation in MSC cultures from 3- and 16-month-old mice. Scale bar, 400 *μ*m. (**f**) Western blot analysis of cleaved-poly ADP-ribose polymerase (PARP) in lysates of trabecular bone dissected from femora of 3- and 16-month-old mice. (**g**) Western blot analysis of Rictor and P-Akt(S473) expression in lysates of trabecular bone from 3- and 16-month-old mice. (**h**) Western blot analysis of mTOR, Rictor, Raptor and P-Akt(S473) expression in lysates of lung, spleen and kidney from 3- and 16-month-old mice. (**i**) Western blot analysis of Rictor, mTOR, Raptor, osteocalcin (OCN) and P-Akt(S473) expression in primary calvarial osteoblasts from 3- and 16-month-old mice

Age-related bone loss phenotypes were characterized in mice. Micro-computed tomography (micro-CT) analyses of the distal femur showed a marked decrease in bone mass in aged mice (16 months old) compared with young mice (3 months old), as demonstrated by a significant decrease in the trabecular bone volume per tissue volume (BV/TV), trabecular number (Tb.N), trabecular thickness (Tb.Th) and bone mass density (BMD), coupled with an increase in trabecular separation (Tb.Sp) (Supplementary Figure 1). Histological and immunohistochemical analyses of femora revealed an increased number of larger area of adipocytes, characterized by fat vacuoles in the bone marrow (Supplementary Figure 2A), and decreased numbers of osteocalcin-positive osteoblasts (Figure 1a) and TRAP-positive osteoclasts on the trabecular and endosteal bone surfaces in aged mice (Supplementary Figure 2B). The bone marrow fat accumulation and decreased osteoblast number implies that there was a switch from osteogenesis to adipogenesis in the BMSCs. Interestingly, scanning electronic microscope (SEM) analyses revealed a rough trabecular bone surface with many attached cells in young mice, but a smooth trabecular bone surface with little cell adhesion in aged mice (Figure 1b). Many empty lacunae or lacunae with crimpled cells (Figure 1b), accompanied with a reduction in bone formation rate (BFR) (Figure 1c), implicating decrease of osteoblast adhesion during aged-related bone loss.

To further confirm these age-related phenotypes *in vitro*, calvarial osteoblasts from 3- and 16-month-old mice were cultured. Osteoblasts from old mice exhibited a significantly reduced capacity for cell adhesion (Figure 1d) and mineralization (Figure 1e), but enhanced apoptosis (Figure 1f). Taken together, these findings suggest that in addition to the switch from the osteogenic differentiation to adipogenic differentiation of BMSCs, decreased osteoblast adhesion and mineralization, and enhanced apoptosis, may have important roles in aged-related osteopenia.

Rictor/mTORC2 has been shown to control both actin cytoskeletal organization and cell survival.^24, 27, 28^ To explore the mechanism responsible for the reduced capacity of cell adhesion and survival as osteoblasts age, trabecular bone samples were dissected from the femora of young and aged mice, and total protein was extracted to examine Rictor expression by western blotting. Interestingly, expression of Rictor and phosphorylation of mTORC2 substrate Akt(S473) were markedly reduced in the trabecular bone of aged mice (Figure 1g). In contrast, neither the level of mTORC1 (mTOR and Raptor) in bone, nor levels of Rictor in other organs including the spleen, kidney and lung, showed any significant changes with aging (Figure 1h), implicating a bone-specific downregulation of Rictor in aged mice. The downregulation of Rictor, but not mTOR or Raptor, was further confirmed in cultured primary calvarial osteoblasts from aged mice (Figure 1i). Accordingly, dephosphorylation of Akt(S473), the downstream target of Rictor/mTORC2, occurred in osteoblasts from aged mice (Figures 1g and i). Thus, we have identified aging-induced specific downregulation of Rictor in osteoblasts, which may have a role in age-related bone loss.

### Deletion of Rictor in osteoblasts impairs bone formation and stimulates age-related bone loss

**Figure 2:**
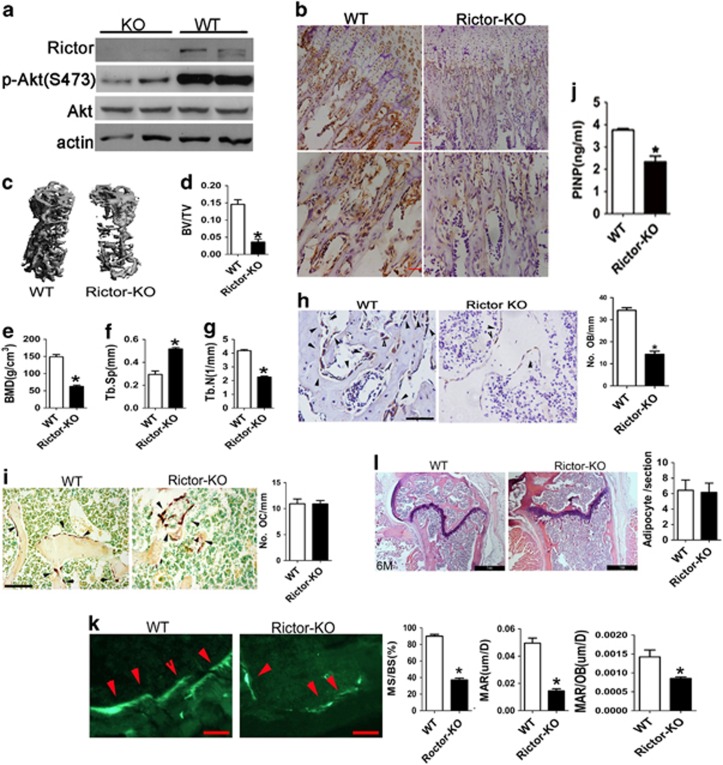
Deletion of Rictor in osteoblasts impairs bone formation and stimulates aged-related bone loss. (**a**) Western blot analysis of Rictor and P-Akt(S473) expression in primary cultured calvarial osteoblasts from OBRictorKO and wild-type mice. (**b**) Representative IHC staining for p-Akt(S473) of distal femora from OBRictorKO and wild-type mice; scale bar, 100 *μ*m. (**c**) Representative micro-CT scans of vertebrae from 6-month-old OBRictorKO and wild-type mice. (**d**–**g**) Changes in vertebral trabecular BV/TV, BMD, Tb.Sp and Tb.N with age in OBRictorKO and wild-type mice (*n*=6, *P<0.01). (**h**) Representative IHC staining for OCN in distal femora from 6-month-old OBRictorKO and wild-type mice (*n*=6). Arrow, osteoblast; scale bar, 50 *μ*m; *P<0.01 between genotypes. (**i**) Representative TRAP staining for osteoclasts of distal femora from 6-month-old OBRictorKO and wild-type mice (*n*=6). NS, no significant difference between genotypes; scale bar, 100 *μ*m. (**j**) Enzyme-linked immunosorbnent assay (ELISA) analysis of serum Procollagen Type I N-terminal propeptide (PINP) levels in 6-month-old OBRictorKO and wild-type mice (*n*=6).*P<0.01 *versus* WT. (**k**) Representative calcein staining and quantification of MS/BS, MAR and MAR/osteoblast in distal femora from 6-month-old OBRictorKO and wild-type mice (*n*=3 *P<0.01 *versus* OBRictorKO). (**l**) Representative HE staining of distal femora from OBRictorKO and wild-type mice, adipocytes were counted (*n*=6). NS, no significant difference between genotypes; scale bar, 1 mm

To investigate the role of Rictor/mTORC2 in osteoblast and bone formation, we generated conditional knockout mice with *Rictor* deletion limited to the osteoblasts by crossing floxed Rictor mice with Osx-GFP-Cre mice (which express a GFP-Cre fusion protein under the direction of the Osx1 promoter). We mated Osx-GFP-Cre^TG/+^ with Rictor^flox/flox^ mice and selected female mice with the genotype Osx-GFP-Cre^TG/+^;Rictor^flox/flox^ (here after referred to as OBRictorKO) for detailed analysis. Female Osx-GFP-Cre^TG/+^;Rictor^+/+^ littermates served as controls. OBRictorKO mice were born at the expected Mendelian frequency, and recombination and deletion of Rictor alleles only occurred in skeletal tissues (i.e., legs and skull) as demonstrated by allele-specific PCR (Supplementary Figure 3) and western blotting (Figure 2a). Immunohistochemical staining of distal femur sections showed a dramatic decrease in P-Akt(S473) in OBRictorKO mice (Figure 2b), indicating that mTORC2 was inactivated by Rictor disruption.

Although no significant differences in body weight and length were observed between Rictor-deficient mice and littermate controls at all ages. OBRictorKO mice developed osteoporosis with increasing age (Figures 2c–g and Supplementary Figure S4). In OBRictorKO mice at 6 months of age, BV/TV fell markedly compared with wild-type littermates (*P*<0.001; Figure 2d and Supplementary Figure S4). Cortical bone thickness (Ct.Ch) was also significantly decreased in Rictor deletion mice (Supplementary Figure S5). Histological analysis of the tibial metaphysis in 6-month-old mice showed that osteoporosis in OBRictorKO mice was associated with a striking decrease in osteoblasts (OCN-positive cells) number per unit of bone surface (Figure 2h), whereas the osteoclast number decreased slightly (Figure 2i). In keeping with these observations, serum levels of the N-terminal propeptide of type 1 procollagen (P1NP), a bone formation marker, were reduced in 6-month-old OBRictorKO mice compared with wild-type mice (Figure 2j). The BFR of 6-month-old OBRictorKO mice was also reduced compared with littermate controls (Figure 2k). In addition, no detectable adipocyte accumulation could be observed in 6-month-old OBRictorKO mice (Figure 2l). These data demonstrate that the age-related osteoporosis in OBRictorKO mice is associated with a reduction in bone formation in the face of normal osteoclast activity, but not an accumulation of bone marrow fat.

### Rictor is essential for osteoblast adhesion, mineralization and survival

**Figure 3:**
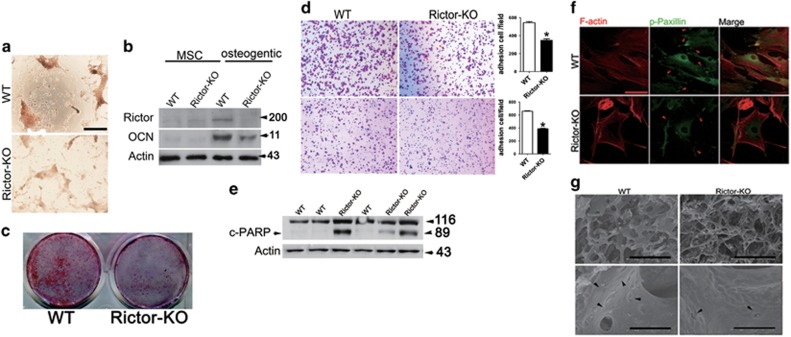
Rictor is essential for osteoblast adhesion, mineralization and survival. (**a**) Photomicrographs of calcium nodule in MSCs isolated from wild-type and OBRictorKO mice after osteognenic induction for 21 days. (**b**) Western blot analysis of Rictor and OCN expression in MSCs from OBRictorKO and wild-type mice that underwent osteogenic induction for 14 days. (**c**) Photomicrographs of bone nodule formation in osteoblast cultures from OBRictorKO and wild-type mice. (**d**) Cell adhesion analysis of primary osteoblast cultures from OBRictorKO and wild-type mice with (lower panel) or without (upper panel) matrigel in the plate (*n*=3). *P<0.01 compared with wild-type mice. (**e**) Western blot analysis of cleaved-PARP expression in trabecular bone lysates from 6-month-old OBRictorKO and wild-type mice. (**f**) Representative photomicrographs of F-actin (red fluorescence) and p-Paxillin (Y118 green fluorescence) expression in primary osteoblasts from OBRictorKO and wild-type mice. Arrow, p-Paxillin (Y118); scare bar, 5 *μ*m. (**g**) SEM analysis of trabecular bone from 6-month-old OBRictorKO and wild-type mice. Upper panel showed less and thinner trabecula bone in OBRictorKO mice. Black arrow, osteoblast or osteocyte; upper panel bar, 500 *μ*m; lower panel bar, 50 *μ*m

In order to investigate the mechanisms underlying the reduced bone formation in OBRictorKO mice, we first explored osteoblast and adipocyte differentiation in MSC cultures prepared from OBRictorKO and wild-type mice. Although deletion of Rictor in MSCs has been reported to induce a switch from osteoblastogenesis to adipogenesis,^25, 26^ BMSCs from OBRictorKO mice did not exhibit increased efficiency in adipogenesis (Supplementary Figure S6). This indicates that deletion of Rictor in osteoblasts did not affect BMSC differentiation, consistent with the unchanged bone marrow fat in OBRictorKO mice. However, BMSCs from OBRictorKO mice had a significantly reduced capacity to form mineralized bone nodules when cultured in osteogenic medium, accompanied by disruption of Rictor at late differentiation stages (Figures 3a and b). Similar results were obtained in cultures of calvarial osteoblasts (Figure 3c). These results suggest that deletion of Rictor in osteoblasts does not reduce osteoblastic differentiation of BMSCs, but inhibits osteoblast bone formation.

To further explain the decreased osteoblast number in the trabecular bone surface of OBRictorKO mice, the cell adhesion capacity and survival of cultured osteoblasts from OBRictorKO and wild-type mice were examined. Osteoblasts from OBRictorKO mice had a significantly reduced capacity to attach to the culture matrix and increased cleaved PARP (Figures 3d and e). Furthermore, phosphorylation of paxillin, a downstream target of Rictor that regulates cytoskeletal organization, was impaired in Rictor-deleted osteoblasts (Figure 3f). Most importantly, SEM analyses revealed diminished cell adhesion on the trabecular bone surface (Figure 3g), replicating the phenotypes seen in the osteoblasts of aged mice. Together, these findings demonstrate that Rictor is essential for osteoblast adhesion, function and survival.

### Rictor is a target of miR-218 in osteoblasts

We next investigated the mechanism underlying the downregulation of Rictor expression in osteoblasts in aged mice. q-PCR analysis showed that Rictor mRNA-level in osteoblasts from aged mice remained unchanged compared with young mice (Supplementary Figure S7), suggesting that transcriptional regulation does not contribute to the downregulation of Rictor in osteoblasts.

**Figure 4:**
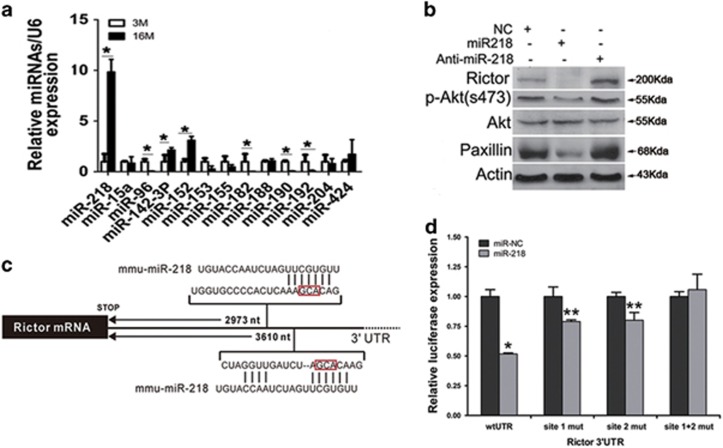
Rictor is a target of miR-218 in osteoblasts. (**a**) Real-time PCR analysis of 13 miRNAs expression in trabecular bone from 3- and 16-month-old mice (*n*=6). *P<0.01 *versus* 3M. (**b**) Western blot analysis of Rictor, p-Paxillin(Y118) and P-Akt(S473) expression in osteoblasts transfected with miR-218 mimics and miR-218 inhibitors. (**c**) The putative miR-218 binding site in the Rictor 3′ UTR. (**d**) Luciferase activity in MC3T3-E1 cells co-transfected with miR-218 mimics or negative control (NC) and the indicated 3′ UTR-driven reporter constructs (*n*=3). *P<0.01 *versus* NC; **P<0.05 *versus* NC

Several microRNAs, including miR-188, miR-218, have been reported to target Rictor in human and mice cells.^29, 30, 31, 32, 33^ Thus, we performed a bioinformatic analysis of potential miRNAs targeting Rictor in mice (Supplementary Table S2). Quantitative real-time PCR analysis of the level of 13 high score miRNAs revealed that miR-218 was strongly upregulated in osteoblasts from aged mice (expression increased 1013% in osteoblasts from aged mice compared with those from young mice) (Figure 4a). Transfection of mimics of miR-218 strikingly downregulated Rictor, whereas miR-218 antagomirs upregulated Rictor protein level (Figure 4b). Accordingly, phosphorylation of Akt(S473) and paxillin, two downstream targets that mediate Rictor-regulated cell survival and actin organization, respectively, were enhanced by miR-218 mimics or reduced by miR-218 antagomirs (Figure 4b). There are two miR-218 sites in the Rictor 3′ UTR (Figure 4c).

To determine whether miR-218 targets Rictor directly in osteoblasts, we cloned the 3′ UTR of Rictor into a luciferase construct. Reporter assays using miR-218-expressing osteoblasts revealed that miR-218 repressed Rictor UTRs (Figure 4d). Mutations of two putative miR-218 sites which are conservedin human *versus* mouse in the Rictor 3′ UTR abrogated responsiveness to miR-218 (Figure 4d). These results suggest that both of these predicted sites contribute equally to miR-218-mediated Rictor reduction. Together, these results suggest that miR-218 directly targets Rictor in osteoblasts.

### miR-218 represses osteoblast adhesion and survival by targeting Rictor

**Figure 5:**
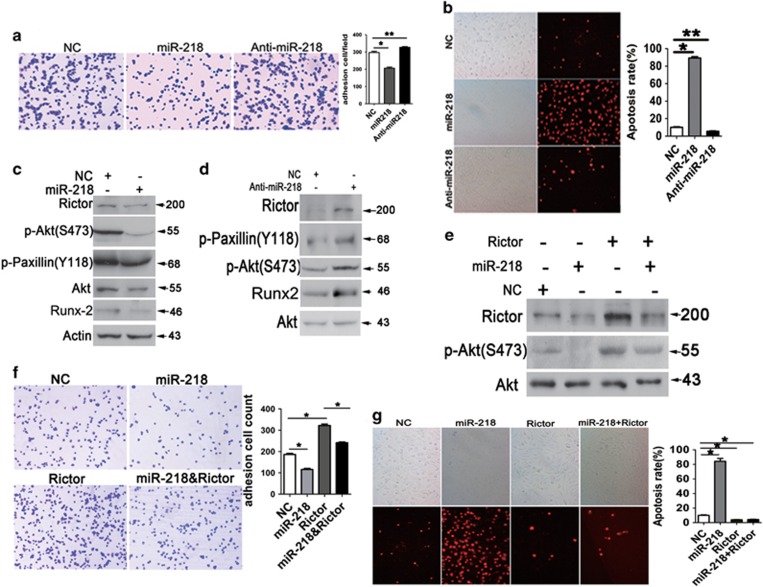
miR-218 represses osteoblast adhesion and survival by targeting Rictor. (**a**) Adhesion analysis of MC3T3-E1 cells transfected with miR-218 mimics, miR-218 inhibitors and NCs (*n*=3). *P<0.01, **P<0.05. (**b**) AnnexinV analysis of apoptosis in MC3T3-E1 cells transfected with miR-218 mimics or miR-218 inhibitor and serum-starved for 48 h (*n*=3). *P<0.01, **P<0.05. (**c**) Western blot analysis of Rictor, p-Paxillin(Y118), Runx2 and p-Akt(S473) expression in MC3T3-E1 cells transfected with miR-218 mimics. (**d**) Western blot analysis of Rictor, p-Paxillin(Y118), Runx2 and p-Akt(S473) expression in MC3T3-E1 cells transfected with miR-218 inhibitor or NCs. (**e**) Western blot analysis of Rictor, p-Paxillin(Y118) and p-Akt(S473) expression in MC3T3-E1 cells transfected with miR-218 mimics and/or Rictor-expressing vectors. (**f**) Adhesion analysis of MC3T3-E1 cells transfected with miR-218 mimics and/or Rictor-expressing vector (*n*=3). *P<0.01, **P<0.05. (**g**) AnnexinV analysis of apoptosis in MC3T3-E1 cells transfected with miR-218 mimics and/or Rictor-expressing vector (*n*=3); *P<0.01, **P<0.05

To further define the role of miR-218 in osteoblasts, the effects of miR-218 mimics and antagomirs on cell adhesion, stress-induced apoptosis in primary osteoblasts and osteoblast cell line MC3T3-E1 were examined. miR-218 mimics reduced cell adhesion and enhanced serum starvation-induced apoptosis, whereas miR-218 antagomirs enhanced cell adhesion, but reduced apoptosis in osteoblasts (Figures 5a and b).

We next asked whether Rictor mediates the regulation of osteoblasts via miR-218. As expected, miR-218 mimics downregulated the Rictor/mTORC2 signaling pathway and phosphorylation of Akt(S473) and paxillin, whereas miR-218 antagomirs enhanced the Rictor/mTORC2 signaling pathway (Figures 5c and d). Importantly, overexpression of Rictor rescued the reduction of Akt(S473) and paxillin phosphorylation and cell adhesion and the enhancement of apoptosis by miR-218 mimics (Figures 5e–g). Together, these findings demonstrate that miR-218 represses osteoblast adhesion and survival by targeting Rictor.

### ROS stimulate miR-218 to downregulate Rictor in osteoblasts from aged mice

**Figure 6:**
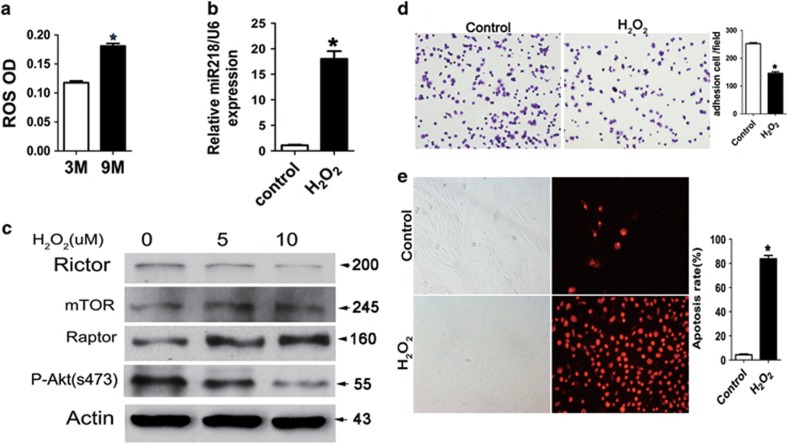
ROS stimulates miR-218 to downregulate Rictor in aged mice osteoblast. (**a**) ROS levels in trabecular bones of 3- and 16-month-old mice (*n*=6); *P<0.01 compared with 3-month-old mice. (**b**) Effect of H_2_O_2_ (1*μ*M) on miR-218 expression in MC3T3-E1 cells (*n*=3). *P<0.01 *versus* controls. (**c**) Western blot analysis of Rictor, mTOR and Raptor expression in MC3T3-E1 cells treated with H_2_O_2_ (5, 10 *μ*M) for 48 h. (**d**) Effect of H_2_O_2_ (1 *μ*M for 24 h) on MC3T3-E1 cell adhesion (*n*=3). *P<0.01 *versus* controls. (**e**) Effect of H_2_O_2_ (10 *μ*M for 36 h) and on MC3T3-E1 cell apoptosis(*n*=3). *P<0.01 *versus* controls

Estrogen deficiency has been considered the primary mechanism of osteoporosis in both women and men, but epidemiological evidence in humans and recent mechanistic studies in rodents indicate that aging and the associated increase in ROS are proximal culprits.^34, 35^ To explain the age-related increase of miR-218 and reduction of Rictor in osteoblasts, we further explored the potential role of ROS in this process. As expected, ROS levels were significantly elevated in the trabecular bone and osteoblast of aged mice (Figure 6a). Strikingly, hydrogen peroxide (H_2_O_2_) treatment increased miR-218 level by 18 times in osteoblasts (Figure 6b; Supplementary Figure S8). Accordingly, Rictor expression, P-Akt(S473) and cell adhesion were reduced, whereas apoptosis was stimulated by H_2_O_2_ (Figures 6c–e). Expression of mTORC1 components, mTOR and Raptor was not affected (Figure 6c), indicating a specific regulation of Rictor/mTORC2 by ROS in osteoblasts. Moreover, previous studies have shown that miR-218 located at 4p15.31 and 5q35.1 within the intron of Slit2 and Slit3.^36^ We found that silencing of either Slit2 and Slit3 decreased the expression of miR-218, suggesting the regulation of miR-218 by its host genes in osteoblasts (Supplementary Figure S9).

### ROS scavenger reduces miR-218 and Rictor expression and aged-related bone loss in mice

**Figure 7:**
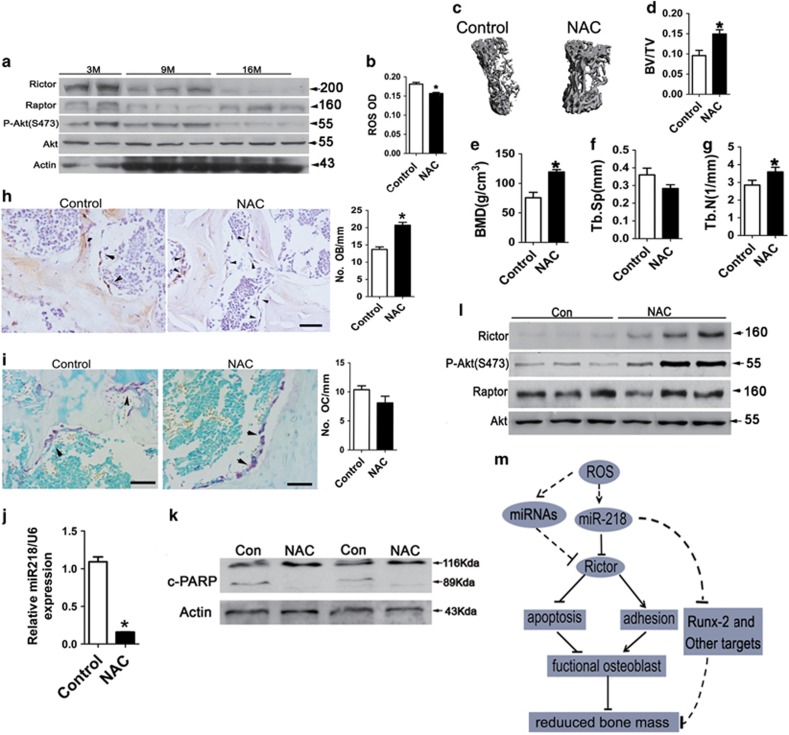
ROS scavenger reduces miR-218 and Rictor expression and aged-related bone loss in mice. (**a**) Western blot analysis of Rictor, Raptor and P-Akt(S473) expression in trabecular bone of 3-, 9- and 16-month-old mice. (**b**) Trabecular bone ROS levels in 9-month-old mice treated with N-acetyl-l-cysteine (NAC; 2 mg/ml) or vehicle for 7 months (*n*=10). *P<0.01 *versus* controls. (**c**) Representative micro-CT scans of vertebrae from mice described in (**b**). (**d**–**g**) Trabecular bone BV/TV, BMD, Tb.Sp and Tb.N in mice described in (**c**) (*n*=10); *P<0.01 *versus* controls. (**h**,**i**) Oestocalcin IHC (**h**) or TRAP (**i**) staining of distal femora from mice described in (**b**). Scale bar, 50 *μ*m; *P<0.01 *versus* controls. (**j**) Real-time PCR analysis of miR-218 expression in trabecular bones of mice described in (**b**)(*n*=10). *P<0.01 *versus* controls. (**k**) Westem bolt analysis of c-PARP expression in trabecular bones of mice described in **(b).** (**l**) Western blot analysis of Rictor, Raptor and P-Akt(S473) expression in trabecular bones of mice described in (**b**). (**m**) A schematic model depicting that the upregulation of miR-218 and loss of Rictor with aging induces the pathogenesis of age-related bone loss

To confirm the correlation of ROS with miR-218 and Rictor expression and aged-related bone loss *in vivo*, aging mice were administrated with ROS scavenger NAC. Because marked bone loss began from the age of 9 months in mice and significant loss of Rictor was also observed in mice at 9 months (Figure 7a), we treated mice at 7 months with NAC to prevent age-related bone loss. We found that in NAC-treated mice (16 months), trabecular bone ROS level was reduced (Figure 7b), whereas BV/TV, trabecular number and trabecular thickness were increased as compared with the control mice (Figures 7c–g; Supplementary Figure S10). Histological and IHC analyses of femora revealed a significant increase in the number of osteocalcin-positive osteoblasts and a slight decrease in the number of osteoclasts in NAC-treated mice (Figures 7h and i). Importantly, in addition to the impaired bone loss, miR-218 level (Figure 7j) and decreased cleaved-PARP, whereas Rictor expression and P-Akt(S473) were enhanced in NAC-treated mice (Figure 7l). These findings suggest that ROS stimulates miR-218-mediated Rictor downregulation in osteoblasts of aged mice.

## Discussion

Our studies demonstrate that downregulation of Rictor with aging in osteoblast has an important role in the pathology of age-related bone loss. Rictor is essential for bone formation by controlling osteoblast cytoskeletal organization, promoting osteoblast adhesion to the bone surface and survival, and eventually maintaining a sufficient number of functional osteoblast cells. We propose a novel functional pathway important for age-related bone loss independent of the accumulation of adipocytes in the bone marrow: accumulation of ROS with aging stimulates transcription of miR-218, which in turn targets Rictor, impairing osteoblast adhesion to the bone surface and promoting osteoblast apoptosis. In consequence, bone formation is reduced, contributing to age-related bone loss (Figure 7m). Meanwhile, other miRNAs (miR-152, miR-142, and so on) could also target to Rictor, and increased miR-218 may have other targets important for osteoblast such as Runx2 in this process (Figure 6c), all of which might contribute to age-related bone loss.

Increased bone resorption and/or decreased bone formation can lead to bone loss, and adecrease in bone formation is believed to be the principal pathogenic mechanism mediating age-related bone loss.^2, 4, 5^ Impaired bone formation may result from an age-related decrease in osteoblast number and function as a consequence of a multitude of intrinsic senescence-related mechanisms.^7^ Because progressive accumulation of fat in the bone marrow space with increasing age has been observed in age-related osteopenia, and bone marrow adipocytes and osteoblasts are thought to originate from common BMSCs, reduction in osteoblastic bone formation is thought to originate from the switch from osteoblast to adipocyte differentiation in BMSCs during aging.^4, 5, 6^ Many molecules including Rictor,^24, 25, 26^ Id4 (12), Maf,^13^ cannabinoid receptor type 1 (ref. 11) and miR-188 (ref. 31) have been shown to act as this molecular switch in BMSC differentiation. However, *in vivo* bone formation depends not only on the number and rate of the generation of osteoblasts, but also on the functional activity of osteoblasts and the lifespan of mature osteoblastic cells. Histomorphometric studies indicate that the age-related decrease in bone mass in mice is associated with increased osteoblastic and osteocytic apoptosis. This study confirms that in cells from aged individuals, more cells were apoptotic. Interestingly, the capacity of osteoblasts from aged mice to adhere to the bone surface was reduced markedly compared with that of young mice both *in vitro* and *in vivo*, which is crucial for the bone formation process. This finding emphasizes the importance of the dysregulation of osteoblast cytoskeletal organization in the pathogenesis of aged-related bone loss. Collectively, decreased osteoblast adhesion on the bone surface and increased osteoblast apoptosis reduce the number of functional osteoblasts during aged-related bone loss.

Recent studies have established the essential role of Rictor/mTORC2 in the differentiation of BMSCs, but the results from *in vitro* and *in vivo* studies are controversial.^24, 25, 26^ Both primary mouse BMSCs deficient in Rictor generated by the Cre/loxP system or BMSCs with Rictor knockdown by siRNA displayed reduced osteogenic differentiation capacity and enhanced adipogenic differentiation potential.^24, 26^ BMSCs from mice with Rictor disruption in the limb skeletogenic mesenchyme (including BMSC) using Prx1-cre also exhibited impaired osteoblast differentiation *in vitro.*^25^ These results demonstrate that mTORC2 regulates cell-lineage selection in favor of osteoblasts over adipocytes *in vitro*. However, osteoblast numbers were surprisingly not reduced, but fewer bone marrow adipocytes were observed in the Rictor-deficient mice, indicating that Rictor/mTORC2 is required for proper bone marrow adipogenesis *in vivo.*^25^ Thus, the function of Rictor in BMSC lineage selection needs to be further clarified. Notably, our *in vitro* and *in vivo* studies demonstrate that Rictor is required for cytoskeletal organization, adhesion, mineralization and survival of differentiated osteoblasts. Our evidence suggests that Rictor has different roles in BMSCs and osteoblasts. Accordingly, loss of Rictor in the limb mesenchyme leads to shorter and narrower skeletal elements in both embryos and postnatal mice. These mice also exhibited impaired bone formation, resulting in thinner cortical bones.^25^ However, deletion of Rictor in osteoblasts in this study did not affect bone growth during development, but accelerated bone loss with increasing age due to deficiency in mineralization, adhesion and survival of osteoblasts, indicating the essential role of Rictor in maintaining a sufficient number of functional osteoblasts and bone formation. Importantly, our study reveals the causal relationship between loss of Rictor and age-related bone loss, suggesting that Rictor downregulation with aging has a significant role in the pathogenesis of age-related osteoporosis.

Many studies have demonstrated that miRNAs have critical roles in all stages of bone formation, suggesting the possibility that miRNAs can be novel therapeutic targets for skeletal diseases.^37^ In BMSCs, miRNA-188 has recently been shown to regulate the age-related switch between osteoblast and adipocyte differentiation, and upregulation of miRNA-188 contributes to aged-related bone loss. Notably, miRNA-188 targets Rictor and leads to its downregulation with aging in BMSCs.^31^ In osteoblasts, we did not observe a significant increase in miR-188 level in aged mice. Instead, enhanced expression of miR-218 targeted Rictor and appeared to be responsible for the loss of Rictor with aging in osteoblasts. miR-218 reduces osteoblast adhesion and enhances osteoblast apoptosis, further confirming the negative role of miR-218 in bone formation. Stage-dependent regulation of Rictor by different miRNAs during osteoblast lineage differentiation highlights the complexity of the regulation of cell differentiation by miRNAs. Both miR-188 and miR-218 are potential therapeutic targets for age-related osteopenia.

In conclusion, this study demonstrates that the downregulation of Rictor with aging in osteoblasts is important for the pathogenesis of age-related bone loss. This pathway is a novel mechanism independent of the switch of BMSCs from osteoblasts to adipocytes and bone fat accumulation. Targeting this pathway, which mediates age-related osteoblastic dysfunction, may be useful to promote bone formation and reduce bone loss associated with aging.

## Materials and Methods

### Materials

All reagents were obtained from Sigma (Poole Dorset, UK) unless otherwise indicated. Antibodies details for western blot analysis and immunohistochemistry can be found in Supplementary Information.

### Mice

All experiments were performed in accordance and approved by the Southern Medical University Experimental Animal Welfare and EthicsCommittee. Mice in which exon 11 of Rictor was flanked with a loxp site (Rictor^flox/flox^) were kindly provided by Professor Mark A. Magnuson at Vanderbilt University.^38^ Osx-GFP-Cre mice (cat#006361) were purchased from Jackson Laboratory (Bar Harbor, ME, USA). We performed genotyping using genomic DNA isolated from tail biopsies, and the primers used are shown in Supplementary Table S1. C57BL/6 mice were purchased from the Laboratory Animal Centre of Southern Medical University. Nine-month-old mice were treated with NAC (oral. 2 mg/ml, dilution with water) for 7 months, *n*=10. Mice were sacrificed by cervical dislocation to ameliorate suffering. Complete Arrive guidelines Checklist is included in Supplementary Table S2.^39^

### Cells culture

The preosteoblast cell line MC3T3-E1 was maintained in alpha-MEM (Gibco BRL, Gaithersburg, MD, USA) supplemented with 10% fetal bovine serum (Gibco), 100 U/ml penicillin and 100 mg/ml streptomycin sulfate. Primary BMSCs and osteoblastic cells were prepared from the bone marrow or calvaria of mice at different ages. For osteogenic induction, 100 *μ*g/ml ascorbic acid (Sigma-Aldrich, St. Louis, MO. Germany) and 10 mM *β*-glycerol phosphate (Sigma-Aldrich) were added to confluent cells. Alizarin red staining was carried out according to standard techniques. Adipogenic medium included 0.5 mM IBMX, 5 *μ*g/ml insulin and 1 *μ*M dexamethasone. Cells were transfected with a Rictor expression vector (pRK5-myc-Rictor, addgene, cat #1860), or with 100 nM miR-218 mimic, miR-218 inhibitor or scramble Control oligo using the transfection agent lipo2000 (Invitrogen, Carlsbad, Canada) according to the manufacturer's instructions.

### Confocal analysis

Osteoblast cells from OBRictorKO and wild-type mice grown on glass coverslips in six-well plates were fixed with 4% ice-cold paraformaldehyde in phosphate-buffered saline (PBS) for 20 min at 4 °C and permeabilized with 0.2% Triton X-100 in PBS for 5 min, then blocked with goat serum for 30 min and incubated in P-Paxillin (Y118) (at 1:100 dilution with 1% BSA) for 1 h. They were then washed in PBS for 3 × 5 min. The cells were stained with FICT-conjugated phalloidin (at 1:500 dilution with 1% BSA) and 488-conjugated secondary antibodies (at 1:250 dilution with 1% BSA) and incubated for 1 h at room temperature. Then, they were again washed in PBS for 3 × 5 min. Photomicrographs were obtained using FLOUVIEW confocal microscopy (Olympus, Tokyo, Japan).

### Cell adhesion assay

Primary osteoblast and MC3T3-E1 cells were trypsinized and resuspended at a concentration of 5 × 10^4^ cells. Cells were allowed to adhere on PLAGA thin films at 37 °C for 1 h. The non-adherent cells were removed by washing with PBS three times and fixed in 70% ethanol for 15 min. The crystal violet stain solution was incubated with the adherent cells for 15 min at room temperature. The stained cells were then counted in a hemacytometer under a light microscope.

### ELISA and ROS assays

Serum PINP was measured using a mouse competitive enzyme immunoassay kit (Elabscience, Shanghai, China), and the concentration of ROS in bone was measured according to the manufacturer's instructions.

### Preparation of decalcified sections, histochemistry and immunohistochemistry (IHC) and histomorphometric analyses

Femur tissues dissected from the mice were fixed using 4% paraformaldehyde in PBS at 4 °C for 24 h and then decalcified in 15% EDTA (pH 7.4) at 4 °C for 14 days. The tissues were embedded in paraffin for optimal cutting temperature compound (Sakura Finetek, Tokyo, Japan), and 2-5 *μ*m sagittal-oriented sections were prepared for histological analyses. H&E staining was routinely performed. Tartrate-resistant acid phosphatase (TRAP) staining was performed using a standard protocol (Sigma-Aldrich). For IHC, we incubated primary antibodies which recognized mouse osteocalcin overnight at 4 °C. All sections were observed and photographed on Olympus BX51 microscope (Tokyo, Japan). Immunohistochemical staining was evaluated by cell number counting. Osteoblasts on bone surface were discerned by morphology and calculated by two independent observers blinded to the groups. At least three mice per group were examined.

### Real-time PCR

Total RNA was extracted from MC3T3-E1 cells using Trizol reagent (Life Technologies, Waltham, MA, USA). TaqMan probes were used for the detection of miRNA (Applied Biosystems, Life Technologies, Waltham, MA, USA) as described by the manufacturer, by using the small nuclear RNA U6 as an endogenous control. For mRNA-level analysis, cDNA was generated by using reverse transcriptase SuperScript II and poly, dTpremers (Invitrogen). Real-time PCR was performed by SYBR Green Master Mix (Invitrogen) and the ABI7500HT fast real-time PCR System (Applied Biosystems). Primers are described in Supplementary Table S1. U6 was used as an endogenous control. The relative quantification of miRNA expression was performed using the ΔΔCT method.

### Vector constructs

The 3′-UTR of Rictor, encompassing the predicted miRNA sequences, was inserted into the multiple cloning site of the reporter vector psiCHECK-2 (Promega, Madison, USA) with *Xho*I and *Not*I. The seed-region-mutated reporters contained engineered point mutations of four nucleotides complementary to the 5′-end of the relevant miRNA. The primers used for cloning are listed in Supplementary Table S1. All sequences were confirmed by Sanger sequencing.

### Luciferase assays

HEK293 cells were transfected with the indicated psiCHECK-2 luciferase construct. All cells were also transfected with miRNA mimics or negative control. Lysates were collected 36 h after transfection and luciferase activities were measured by using the dual-luciferase reporter assay system (Promega).

### miRNA detection

Total RNA, inclusive of the small RNA fraction, was extracted from cultured cells with a Tissue Total RNA Extraction Kit (GenePharma). Reverse transcription reactions were carried out using Moloney murine leukemia virus reverse transcriptase (M-MLV RT) (Invitrogen). Real-time PCR was performed on an Applied BiosystemStepOnePlus, using SYBR Green I Real-Time PCR kits (GenePharma, Suzhou, China) for miRNA. The relative expression levels of miRNAs in each sample were calculated and quantified using the 2^−total^ method after normalization for expression of the positive control.

### Scanning electron microscopy (SEM)

The surface of the methylmethacrylate (MMA) embedded femurs were polished and acid-etched with 37% phosphoric acid for 2–10 s. After washing for 5 min with 5% sodium hypochlorite they were coated with gold and palladium before examining with SEM (S-3700 N, Hitachi, Tokyo, Japan).

### Calcein staining

Three- and nine-month-old mice were given calcein via intraperitoneal injection,10 and 3 days before killing. Both femur were fixed in 100% ethanol, embedded in polymethyl methacrylate and sectioned using microtome (Leica SM2500S; knife: Leica VMH400, Nussloch, Germany). A total of 10 *μ*m thick longitudinal section from each femur was analyzed using Olympus microscope. The flourochrome labels were used to assess the bone MARs. We calculated MAR by measuring the mean distance of fluorescent labels, and dividing the distance by the time point at which the labels were administrated. MAR/OB: average thickness of mineral apposition by an osteoblast per day in 1 mm bone surface.

### micro-CT analysis

Quantitative analysis was performed in mice femora at 12-*μ*m resolution on a micro-CT Scanner (Scanco Medical, Bassersdorf, Switzerland). Briefly, scanning was performed at the lower growth plate in the femora and extended proximally for 300 slices. We started morphometric analysis with the first slice in which the femoral condyles were fully merged and extended for 100 slices proximally. The 3D structure and morphometry were constructed and analyzed for BV/TV (%), BMD (mg HA/mm^3^), Tb.N. (mm^–1^), Tb.Th. (mm) and Tb.Sp. (mm). We also performed micro-CT imaging in the mid-diaphysis of the femur and performed mid-shaft evaluation of 100 slices to quantify the cortical thickness, bone mineral density and outer/inner perimeter of the mid-shaft.

### Trabecular bone ROS assay

Trabecular were isolated from femur and ground with liquid nitrogen. The homogenate in PBS was centrifuged to remove debris and the supernatants were incubated in 10 *μ*M dichorodihydro fuorescein diacetate (DCFH-DA; Molecular Probes, GenePharma, Suzhou, China) for 10 min at room temperature. DCFH-DA oxidation into 2′,7′-dichlorofluorescein was measured using a spectrofluorometer (Bio-TEK, Vermont, USA, excitation 485 nm and emission 525 nm). Date were expressed as value of optical density (OD).

### Western blot

Cells and tissues were lysated by 2% sodium dodecyl sulfate with 2 M urea, 10% glycerol, 10 mM Tris–HCl (pH 6.8), 10 mM dithiothreitol and 1 mM phenylmethylsulfonyl fluoride. The lysates were centrifuged and the supernatants were separated by SDS–PAGE and blotted onto a nitrocellulose (NC) membrane (Bio-Rad Laboratories, Shanghai, China). The membrane was then analyzed using specific antibodies and visualized by enhanced chemiluminescence (ECL Kit, Amersham Biosciences, GE Healthcare, Buckinghamshire, UK).

### Preparation of undecalcified histology sections

To label the mineralization fronts, 6-month-old mice were subcutaneously injected with calcin (Sigma, 15 mg/kg body weight) in 2% sodium bicarbonate solution 10 and 3 days before death. After dissection, the femurs were fixed in 4% paraformaldehyde for 24 h. They were then dehydrated through a graded series of ethanol (70–100%) and xylene before being embedded in MMA without prior decalcification. Ten micrometer-thick sections were prepared for double-labeling fluorescent analysis.

### Statistics

All results are expressed as mean±S.E.M. T-tests were used to calculate *P*-values. Statistical significance was defined as *P*<0.05.

## Abbreviations

BMD: bone mineral density
BMSCs: bone marrow mesenchymal stem cells
BV/TV: bone volume per tissue volume
MSC: mesenchymal stem cell
mTORC2: mechanistic target of rapamycin complex 2
Tb.N: trabecular number
Tb.Sp: trabecular separation
Tb.Th: trabecular thickness
TRAP: tartrate-resistant acid phosphatase
BS: bone furface
NAC: N-acety-l-cysteine
ROS: reactive oxygen species

## Supplementary Material

Supplementary Informations
