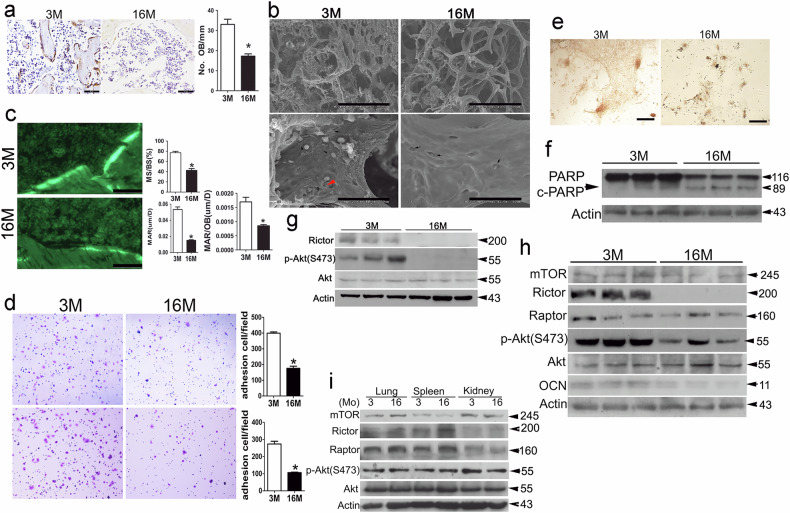# Correction: Loss of Rictor with aging in osteoblasts promotes age-related bone loss

**DOI:** 10.1038/s41419-026-08784-0

**Published:** 2026-05-13

**Authors:** Pinling Lai, Qiancheng Song, Cheng Yang, Zhen Li, Sichi Liu, Bin Liu, Mangmang Li, Hongwen Deng, Daozhang Cai, Dadi Jin, Anling Liu, Xiaochun Bai

**Affiliations:** 1 Academy of Orthopedics in Guangdong Province, The Third Affiliated Hospital of Southern Medical University, Guangzhou, 510630 China; 2 State Key Laboratory of Organ Failure Research, Department of Cell Biology, School of Basic Medical Sciences, Southern Medical University, Guangzhou, 510515 China; 3 Department of Biochemistry, Institute of Genetic Engineering, Southern Medical University, Guangzhou, 510515 China; 4 Department of Spine Surgery, The Third Affiliated Hospital, Sun Yat-Sen University, Guangzhou, 510630 China

Correction to: *Cell Death & Disease* 10.1038/cddis.2016.249, published online 13 October 2016

Upon a thorough re-examination of the original raw data and the doctoral dissertation of the first author, Dr. Pinglin Lai, we have identified inadvertent errors in the published version of the article. Specifically, inaccuracies were found in the image labeling and data presentation in Figures 1a, 1h, and 3e, along with spelling errors in Figure 8m. We confirm that the corrected data accurately represents the experiment and does not alter the conclusions of this study.

Incorrect figure
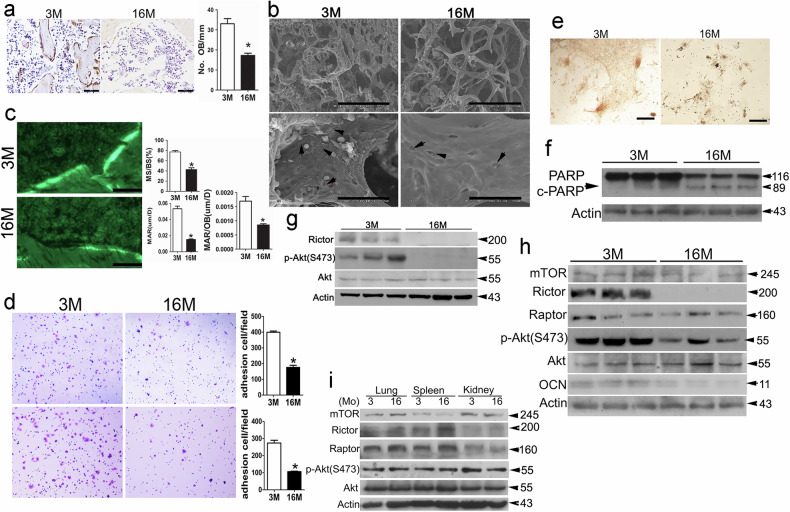


Correct figure