# Supplementary material for: Loss of Rictor with aging in osteoblasts promotes age-related bone loss
Source: Cell Death Dis. 2016 Oct 13;7(10):e2408–. doi: 10.1038/cddis.2016.249 (PMC5133960; doi:10.1038/cddis.2016.249)
Supplement: Supplementary Informations [file cddis2016249x1.docx]

**Supplementary Information**

**Supplementary Materials and Methods**

**Antibodies**

For Western blot analysis and immunohistochemistry, antibodies for Phospho–Paxillin(Y118) (#612405, dilution 1：1000 in immunoblotting and 1:100 in immunohistochemistry), Rictor (#9476, dilution 1:1000 in immunoblotting), Akt (#C67E7, dilution 1:2000 in immunoblotting) were from Cell Signaling Technologies. OCN (#ab93876, dilution 1:1000 in immunoblotting and 1:200 in immunohistochemistry) was from Abcam.Runx-2 (#sc10759,dilution 1:1000 in immunoblotting) were from Santa Cruz Biotechnology. Phospho-Akt (Ser473) (#sc-7985-R, dilution 1:1000 in immunoblotting) and mTOR(#sc1549, dilution 1:500 in immunoblotting) were from Santa Cruz. For the loading control, we used antibodies to β-actin (#M20010, dilution 1:10000 in immunoblotting) from Abmart.Raptor (#1689-1, dilutiong 1:1000 in immunoblotting) from Epitomics.

**Oligonucleotides used in this study**
The sequences of oligonucleotides including miRNA mimics and inhibitors, siRNAs, gene cloning and mutagenesis primers were summarized in Supplementary Table S1.

**Table S1. Oligonucleotides used in this study**

| **Sequence Name** | **Sequence (5’ to 3’)** |
| --- | --- |
| **PCR primers** |  |
| *Osx-GFP-Cre* F | CTCTTCATGAGGAGGACCCT |
| *Osx-GFP-Cre* R | GCCAGGCAGGTGCCTGGACAT |
| *Rictor^loxp/loxp^* F1 | GAAGTTATTCAGATGGCCCAGC |
| *Rictor^loxp/loxp^* R | ACTGAATATGTTCATGGTTGTG |
| **miRNA mimics and inhibitors** |  |
| RNA duplex control (sense) | UUCUCCGAACGUGUCACGUTT |
| mmu-miR-218 mimics (sense) | UUGUGCUUGAUCUAACCAUGU |
| mmu-miR-188 mimics (sense) | CAUCCCUUGCAUGGUGGAGGG |
| mmu-miR-142-3p mimics (sense) | UGUAGUGUUUCCUACUUUAUGGA |
| mmu-miR-152-3p mimics (sense) | UCAGUGCAUGACAGAACUUGG |
| miRNA inhibitor NC | CAGUACUUUUGUGUAGUACAA |
| mmu-miR-218 inhibitor | ACAUGGUUAGAUCAAGCACAA |
| **The primers for mRNA** |  |
| SLIT2 for | TCTTGGGACTGCGAAGCTAT |
| SLIT2 rev | TTTTTATGTCAACTGCCCAC |
| SLIT3 for | GCGCCTGAACAAGAATAAGC |
| SLIT3 rev | GATCCCCTGGATCTGGTTTT |
| Rictor for | CTGCAGAATGGATGTTGTGTTAGC |
| Rictor rev | TGTTACGGCTCATTCACAATGC |
| **3’UTR cloning Primer** |  |
| Rictor3’UTR for | CCGCTCGAGC GAGACATCCCACAAGACAG |
| Rictor3’UTR rev | ATTTGCGGCCGCT TATGCTTCAGGAGGCTTTA |
| **3’UTR mutagenesis Primer** |  |
| Rictor site 1 for | GCCCCACTCAAATTTCAGACCATTAAG |
| Rictor site 1 rev | CTTAATGGTCTGAAATTTGAGTGGGGC |
| Rictor site 2 for | AGGTTGATCTATTTCAAGATGCAAAG |
| Rictor site 2 rev | CTTTGCATCTTGAAATAGATCAACCT |
| **Silt2 and Silt3 siRNA** |  |
| Silt2-mus-5701 | GCCCUAAAGUCAUCACCAATT |
| Silt3-mus-259 | CUGGACAGAAAUAACAUCATT |

Table S2. Potential miRNAs targeting Rictor

| MicroRNA | Target | Is it conserved human versus mouse ?(Yes/No) | Predicted microRNA |
| --- | --- | --- | --- |
| MiR-218 | Rictor | Yes | √ |
| MiR-142-3p | Rictor | Yes | √ |
| MiR-152 | E2F3\MEF\Rictor | Yes | √ |
| miR-188 | HDAC9 \Rictor | Yes | √ |
| MiR-15a | Rictor | Yes | √ |
| miR-155 | Rictor\RPSKB2 | Yes | √ |
| MiR-24 | Unreported | No (not predict in human) |  |
| MiR-424 | Unreported | Yes | √ |
| MiR-21 | Unreported | No(not predict in human and mouse) |  |
| MiR-144 | mTOR | No(not predict in mouse) | √ |
| MiR-182 | Unreported | Yes | √ |
| MiR-204 | Unreported | Yes | √ |
| MiR-96 | Unreported | Yes | √ |
| MiR-34a | Unreported | No(not predict in human and mouse) |  |
| MiR-153 | P15-Luc p21-Luc | Yes | √ |
| MiR-190 | Unreported | Yes | √ |
| MiR-192 | Unreported | Yes | √ |
| MiR-365 | Unreported | No(not predict in human and mouse) |  |
| MiR-503 | CCND1 | No(not predict in human) |  |

**Supplementary Figures**

**Supplemental Fig.S1.** Representative micro-CT scans from 3-month-old and 16-month-old male mice and changes in trabecular BV/TV, Tb.N, BMD, Tb.Th and Tb.Sp with age(n=6). **P*<0.01 versus 3M.

**Supplemental Fig.S2**.**(A).**Repersentative H&E staining of distal femur in 3 and 16-month-old mice, vacuole left by adipocyte were numbered (n=6).Scale bar, 1mm; *p<0.01 versus 3M. **(B).**Representative photomireographs of the TRAP staining in distal femur in 3 and 16-month-old mice, osteoclast were numbered(n=6). NS, no significant difference; scale bar 200µm.

**Supplemental Fig.S3**.PCR analysis of genomic DNA isolated from wild-type and OBRictorKO mice. Genotyping the F2 generation after intercrossing transgenic OSX-GFP-Cre and loxp mice. Expected band of mutant carrying floxed Rictor allele is 554bp, and mutant carrying an OSX-GFP-Cre recombinase gene under the control of OSX-GFP-Cre promoter is 210bp.

**Supplemental Fig.S4.** Representative micro-CT images (A) and (B-E) quantification the BV/TV, BMD, Tb.N and Tb.Sp of femur from WT and OBRictorKO mice at 6- months-old.

**Supplemental Fig.S5.** Representative micro-CT images (A) and quantification the cortical bone thickness (Ct.Th) (B) and bone mineral density (BMD) (C) of femur from WT and OBRictorKO mice at the age 6-months, (n=6) **P*<0.01.

**Supplemental Fig.S6.** Adipocyte formation in MSCs which were isolated from wild-type and OBRictorKO mice cultured in adipogenic medium stained with Nile red O, adipocyte were numbered (n=6).

**Supplemental Fig.S7.**Expression of Rictor mRNA assessed by quantitative PCR in bone from 3-month-old and 16-month-old mice(n=6).

**Supplemental Fig.S8.** Effect of H_2_O_2_ (1µM) on 13 miRNAs expression in MC3T3-E1 cells; n=3; **P*<0.01, versus controls.

**Supplemental Fig.S9.** The effect of silencing SLIT2 or SLIT3 on the expression of miR-218. MC3T3-E1 cells were transfected with SLIT2 or SLIT3 siRNAs for 60 h and the levels of SLIT2 (A) and SLIT3 (B) mRNA and miR-218 (C) were detected by qPRC. n=3;*P<0.01, versus NC (negative control).

Table 2. Definition and Description of 3D Outcomes for Trabecular Bone Microarchitecture

Abbreviation Variable Description Standard unit

TV Total volume Volume of the entire region of interest mm

3

BV Bone volume Volume of the region segmented as bone mm

3

BS Bone surface Surface of the region segmented as bone mm

2

BV/TV Bone volume fraction Ratio of the segmented bone volume to the total volume of

the region of interest

%

BS/TV Bone surface density Ratio of the segmented bone surface to the total volume of

the region of interest

mm

2

/mm

3

BS/BV Specific bone surface Ratio of the segmented bone surface to the segmented

bone volume

mm

2

/mm

3

Conn.D Connectivity density A measure of the degree of connectivity of trabeculae

normalized by TV

1/mm

3

SMI Structure model index An indicator of the stucture of trabeculae;

SMI will be 0 for parallel plates and 3 for cylindrical rods

(72)

Tb.N Trabecular number Measure of the average number of trabeculae per unit length 1/mm

Tb.Th Trabecular thickness Mean thickness of trabeculae, assessed using direct 3D methods mm

Tb.Sp Trabecular separation Mean distance between trabeculae, assessed using direct 3D

methods

mm

Tb.Th.SD Standard deviation of

trabecular thickness

Measure of the homogeneity of trabecular thickness, assessed

using direct 3D methods

mm

Tb.Sp.SD Standard deviation of

trabecular separation

Measure of the homogeneity of trabecular separation, assessed

using direct 3D methods

mm

DA Degree of anisotropy 1 ¼ isotropic, >1 ¼ anisotropic by definition; DA ¼ length of

longest divided by shortest mean intercept length vector

a

MIL Mean intercept length Measurements of structural anisotropy

Table 2. Definition and Description of 3D Outcomes for Trabecular Bone Microarchitecture

Abbreviation Variable Description Standard unit

TV Total volume Volume of the entire region of interest mm

3

BV Bone volume Volume of the region segmented as bone mm

3

BS Bone surface Surface of the region segmented as bone mm

2

BV/TV Bone volume fraction Ratio of the segmented bone volume to the total volume of

the region of interest

%

BS/TV Bone surface density Ratio of the segmented bone surface to the total volume of

the region of interest

mm

2

/mm

3

BS/BV Specific bone surface Ratio of the segmented bone surface to the segmented

bone volume

mm

2

/mm

3

Conn.D Connectivity density A measure of the degree of connectivity of trabeculae

normalized by TV

1/mm

3

SMI Structure model index An indicator of the stucture of trabeculae;

SMI will be 0 for parallel plates and 3 for cylindrical rods

(72)

Tb.N Trabecular number Measure of the average number of trabeculae per unit length 1/mm

Tb.Th Trabecular thickness Mean thickness of trabeculae, assessed using direct 3D methods mm

Tb.Sp Trabecular separation Mean distance between trabeculae, assessed using direct 3D

methods

mm

Tb.Th.SD Standard deviation of

trabecular thickness

Measure of the homogeneity of trabecular thickness, assessed

using direct 3D methods

mm

Tb.Sp.SD Standard deviation of

trabecular separation

Measure of the homogeneity of trabecular separation, assessed

using direct 3D methods

mm

DA Degree of anisotropy 1 ¼ isotropic, >1 ¼ anisotropic by definition; DA ¼ length of

longest divided by shortest mean intercept length vector

a

MIL Mean intercept length Measurements of structural anisotropy

**Supplemental Fig.S10.** Representative micro-CT images (A) and (B-E) quantification the BV/TV, BMD, Tb.N and Tb.Sp of femur from 9-month-old mice treated with N-acetyl-L-cysteine (NAC; 2mg/ml) or vehicle for 7 months (n=10), **P*<0.01, versus controls.
