# Supplementary figures and images for: Loss of Rictor with aging in osteoblasts promotes age-related bone loss
Source: Cell Death Dis. 2016 Oct 13;7(10):e2408–. doi: 10.1038/cddis.2016.249 (PMC5133960; doi:10.1038/cddis.2016.249)

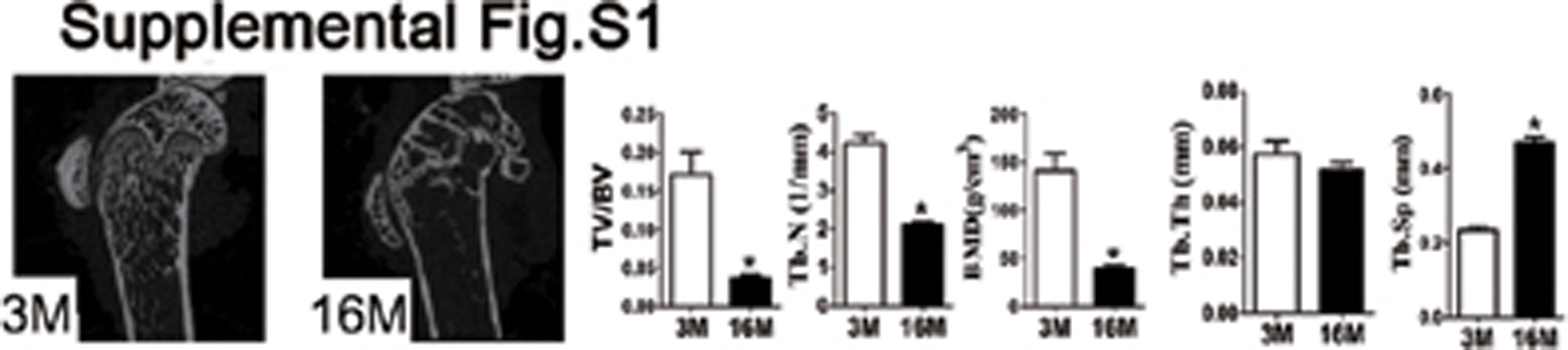

Supplement: Supplementary Figure 1 [file cddis2016249x2.tif]

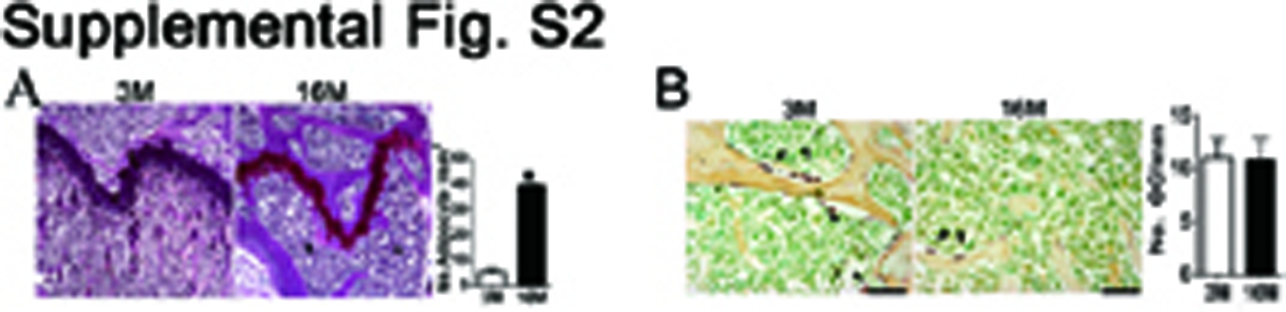

Supplement: Supplementary Figure 2 [file cddis2016249x3.tif]

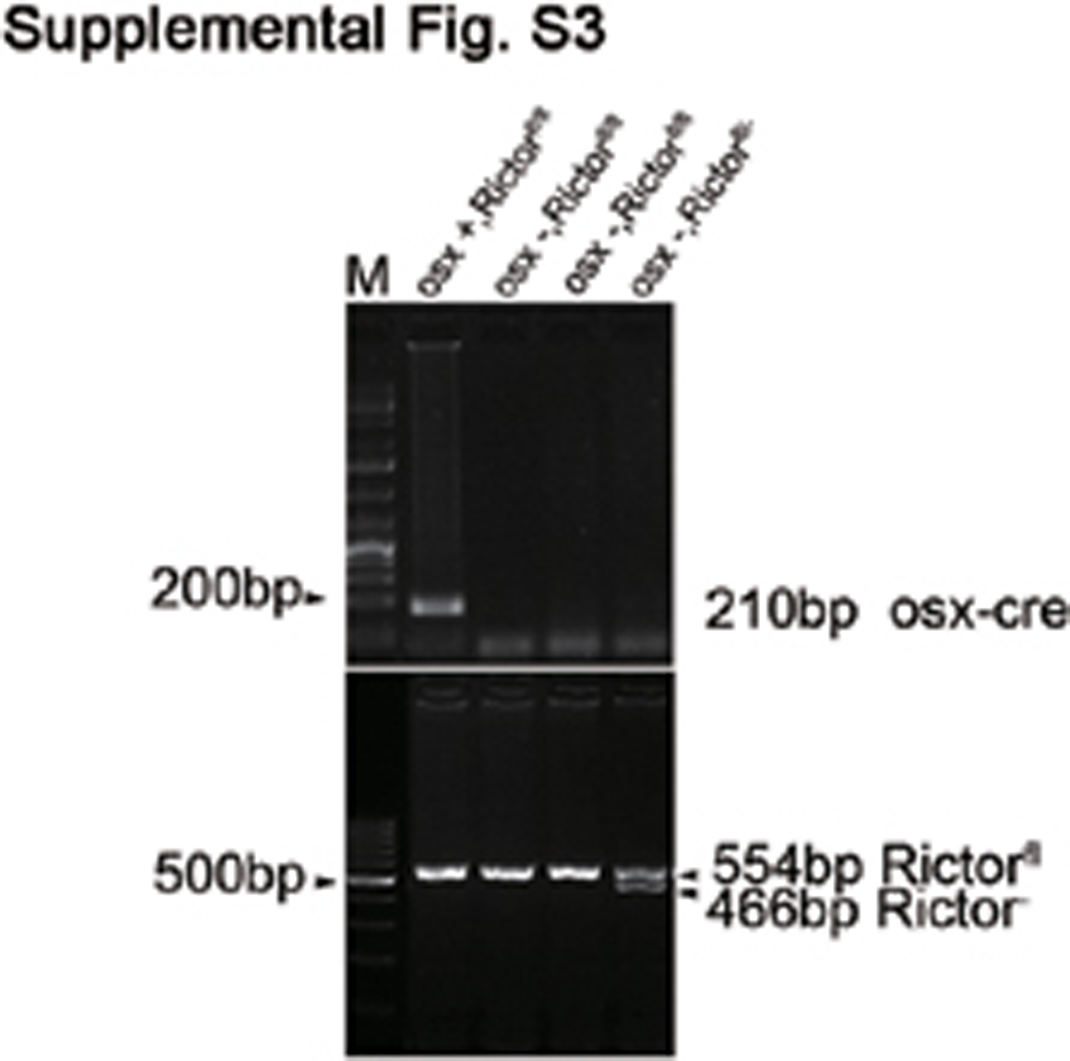

Supplement: Supplementary Figure 3 [file cddis2016249x4.tif]

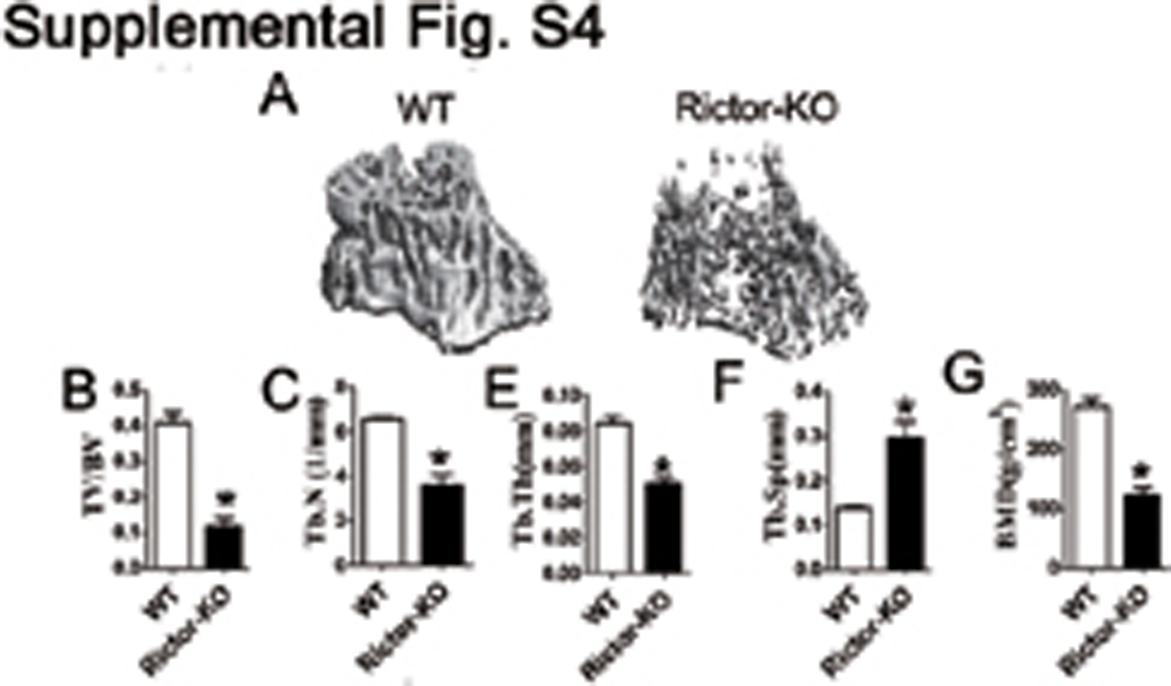

Supplement: Supplementary Figure 4 [file cddis2016249x5.tif]

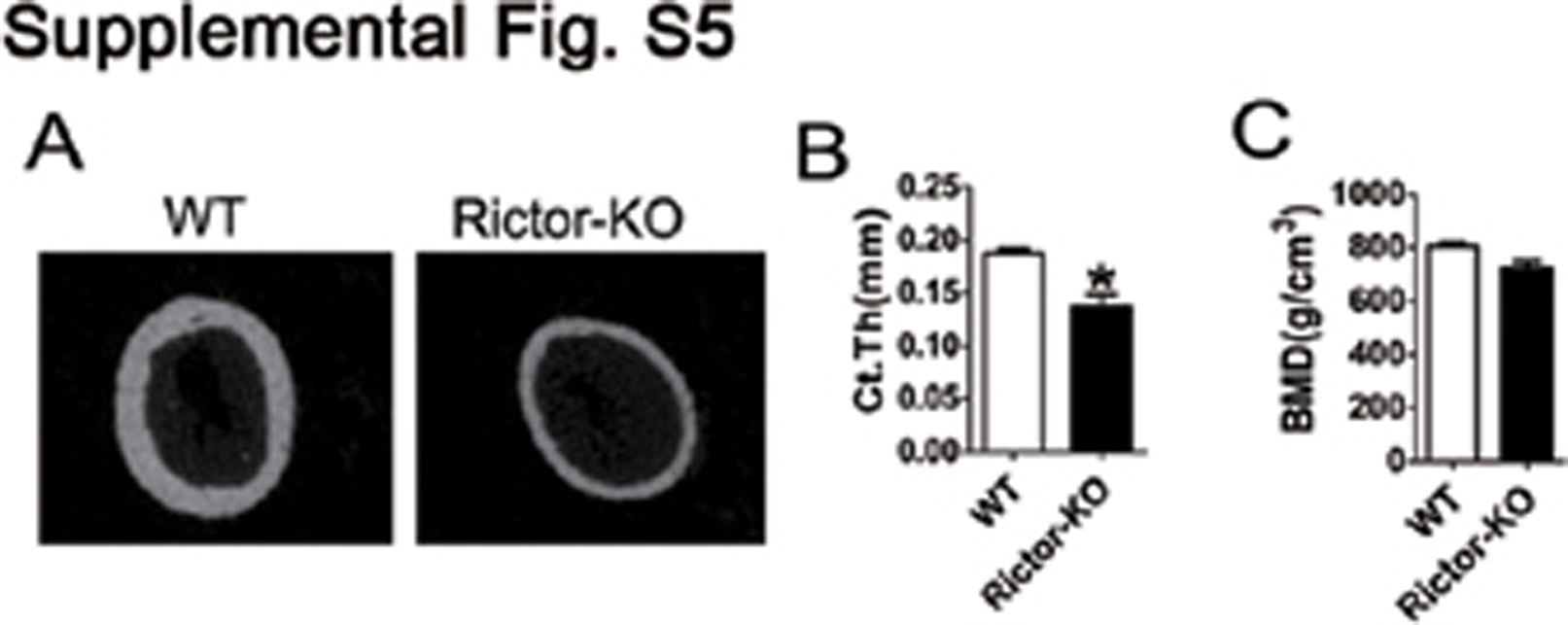

Supplement: Supplementary Figure 5 [file cddis2016249x6.tif]

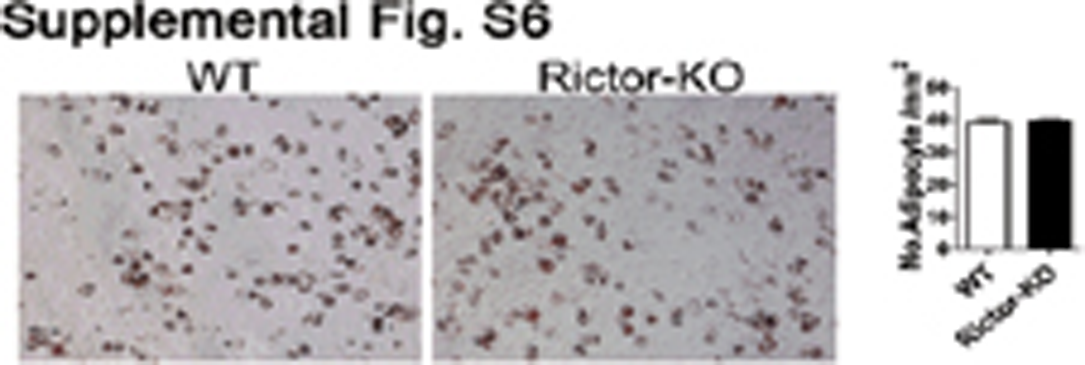

Supplement: Supplementary Figure 6 [file cddis2016249x7.tif]

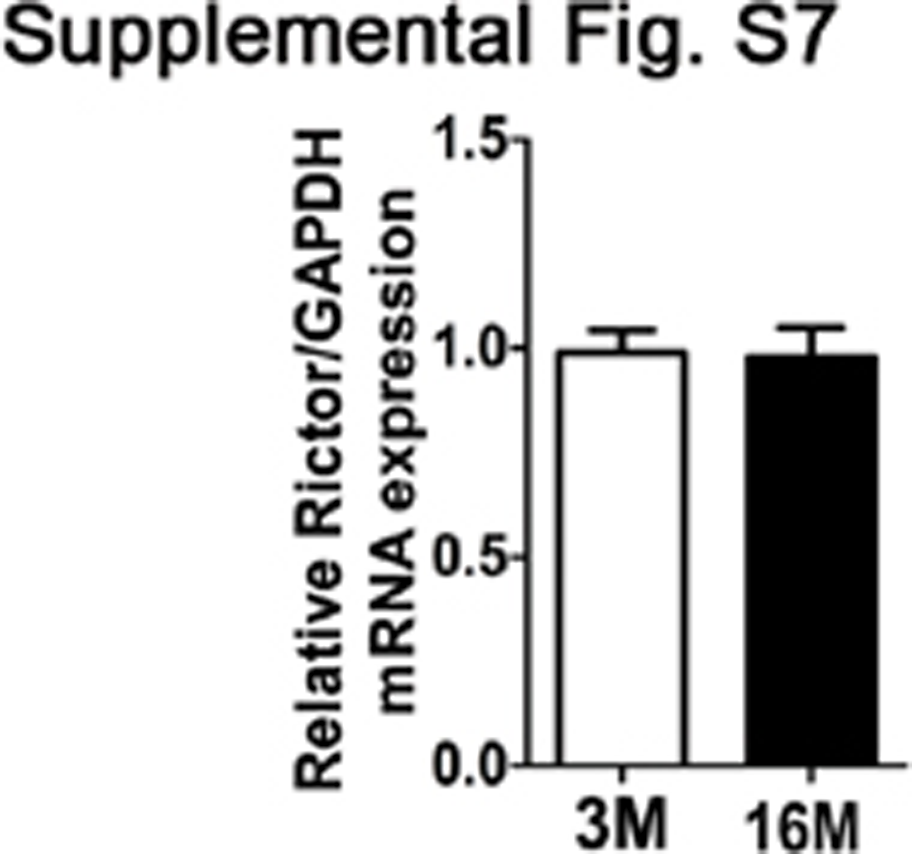

Supplement: Supplementary Figure 7 [file cddis2016249x8.tif]

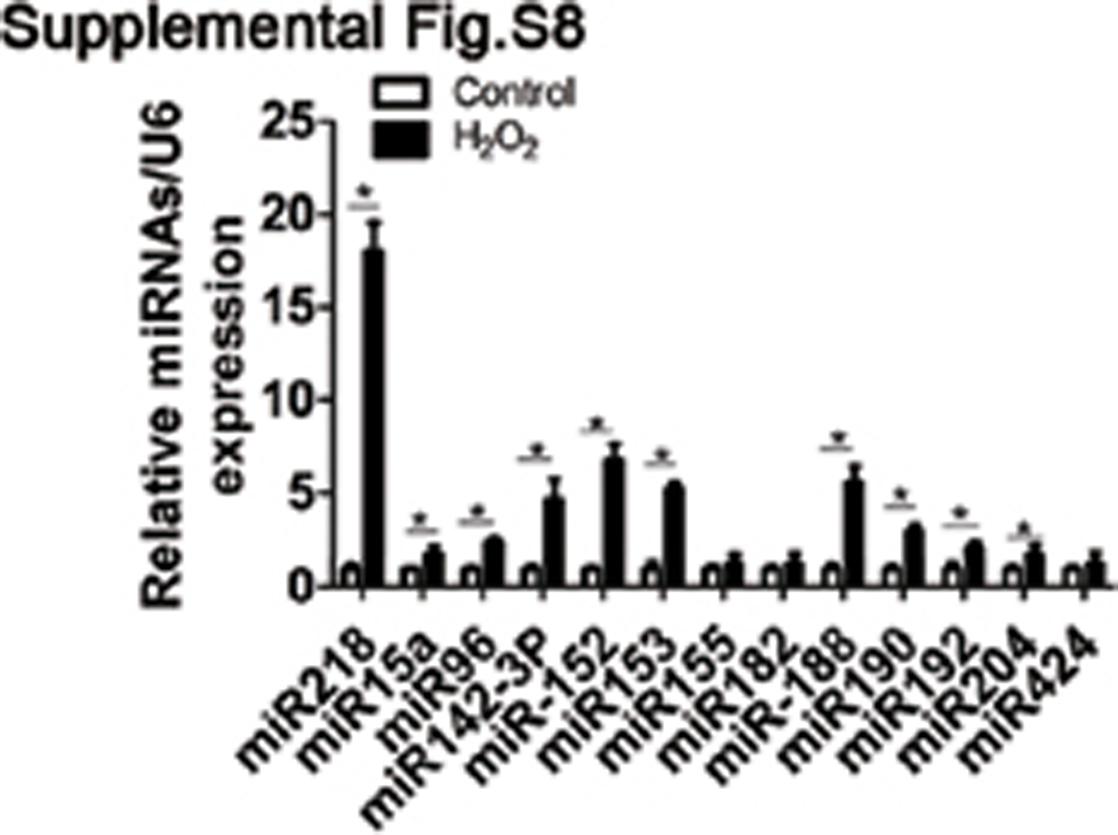

Supplement: Supplementary Figure 8 [file cddis2016249x9.tif]

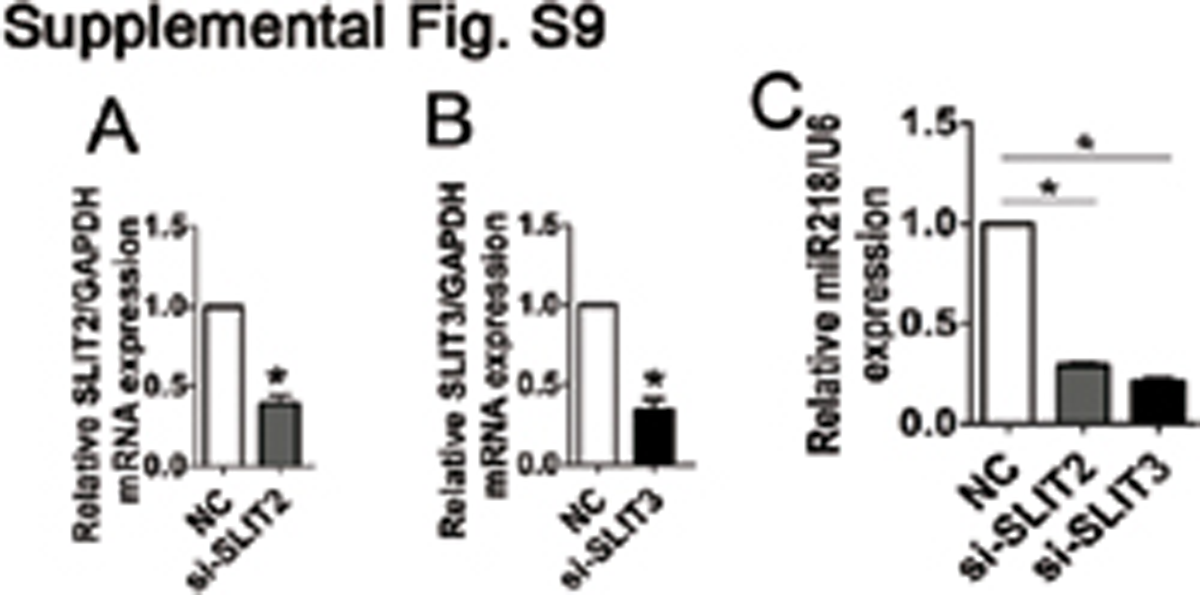

Supplement: Supplementary Figure 9 [file cddis2016249x10.tif]

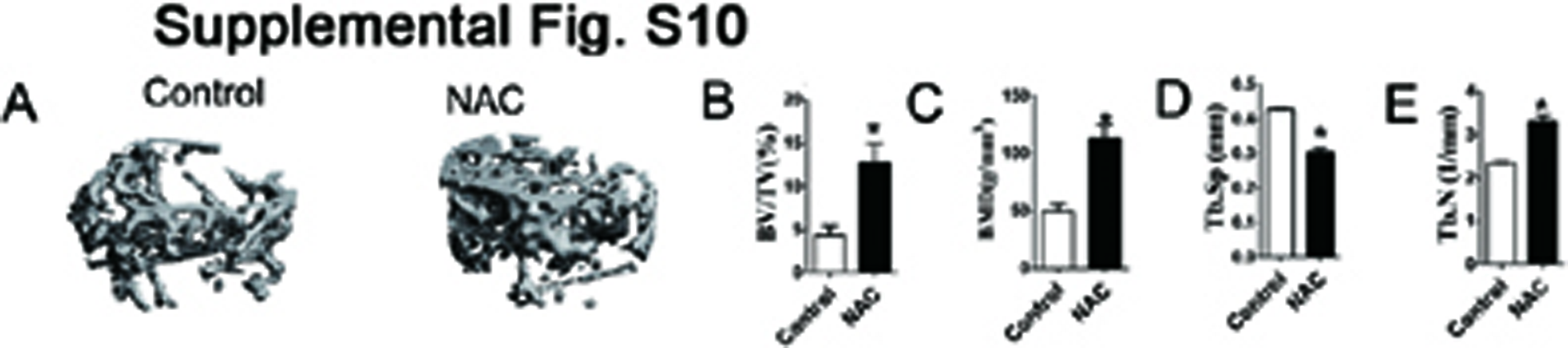

Supplement: Supplementary Figure 10 [file cddis2016249x11.tif]
